# Gleanings from Our Exchanges

**Published:** 1872-06-15

**Authors:** 


### Gleanings from Our Exchanges.

### NOTES ON CANTHARIDES AND A BLISTERING LIQUID.

BY EDWARD R. SQUIBB, M.D.

In a recent revision of the Army Medical Supply Table, the writer was consulted upon the adoption for army use of some of the various blistering-papers, tissues, or plasters, which appear to be so convenient in use, and thus so well adapted to a military organization. Upon careful inquiry, however, it appeared that all these tissues were liable to deterioration under unknown conditions; and that any such want of permanency overbalanced the advantages, particularly for army outposts, where supplies could not be frequently renewed. The tissues were therefore abandoned for the present; but it was thought by the writer that a blistering liquid might be made by taking advantage of the more recent researches upon cantharides, which would give better promise of permanence. Blisters have, in the condition of the patient, and of the surface to which they are applied, and in the degree of skill with which they are applied, so many elements of uncertainty, that it is highly important that the substance used for blistering should be in itself as free as possible from uncertainty. Under different conditions of the patient, a thoroughly active blistering substance may vesicate in four hours, or in seven or eight hours, or may not vesicate at all; and in case of delay or failure, the time is always lost, while it is generally impossible to know whether the cause of delay or failure be in the patient or in the blistering substance.

The general drift of recent observations seems to point to two principal causes of uncertainty in blistering agents. First, the almost absolute insolubility of the active principles of cantharides in ordinary menstrua; and second, the liability of these active principles to become inert, or to fly off in some volatile combination.

The change which takes place in cantharides and its preparations seems somewhat analogous to that which occurs in ergot, and it therefore seems not unlikely that it may be controlled in the same way as that so successfully applied by Prof. Procter in the officinal fluid extract of ergot; that is, by fixing the active principles in new and more permanent combinations.

The important investigation of cantharides in 1852 by Prof. Procter, has served as a basis for many able researches, and notably those of Fumouze, and of Massing and Dragendorff, in 1867, and Delpech in 1870, so that now the older labors of Robiquet and others are rather matters of history than guides in practical application.

In an attempt to utilize still further the labors of these recent writers on cantharides, it was determined to make a solution of cantharadin in some convenient menstruum better than collodion, and to make this so strong as to be as nearly certain as possible. A hundred and twenty pounds of good cantharides was freshly powdered, and the powder carefully assayed yielded the full proportion of 0.466 per cent. of cantharidin. Twenty pounds of this powder very thoroughly exhausted by sixty-eight pounds of chloroform by percolation, and the chloroform recovered by distillation, left a black, oily residue, weighing twenty-eight ounces, avoirdupois. This, while warm, treated repeatedly and copiously with bisulphate of carbon to remove the oily matters, left a residue of very light-colored cantharidin, which in proportionate quantity came but little short of the assay. The bisulphate of carbon distilled off should have left the oily and waxy matters free from cantharidin, but upon trial on the writer's arm, this oil proved to vesicate most promptly and actively. Another portion of this oil, separated from the cantharides in another way, proved equally active. Supposing this activity to be due to its still holding some cantharidin in solution, the oil was repeatedly heated and washed with a solution of potassa so dilute as not to saponify the oil, yet strong enough to render the cantharidin soluble, and to wash it out. Still the oil blistered as actively as before, and the writer was thus forced to the conclusion either that this oil held a portion of the cantharidin too obstinately for easy practical separation, or that the oil itself was vesicant, and therefore that the cantharidin was not, as heretofore believed, the only active or vesicating principle of cantharides. This latter explanation is that which the writer is now inclined to accept in preference to the former.

Rejecting the oil and its vesicating properties, however, it was next thought to get the cantharidin into a definite solution which could be made uniform and thus far trustworthy. A variety of menstrua were tried, including most of those which have been commonly suggested, and glycerin and chloroform and their mixtures in addition. None of these proved to be sufficient solvents, or properly applicable, thus realizing the observations of Prof. Procter as to the peculiar intractability and unmanageable nature of this substance when separated. Many of the cold solutions were vesicant, but all appear to be sluggishly so, and much less active than the oil. These trials all seemed to strengthen the conclusions of Massing and Dragendorff that cantharidin is an anhydride, which in combination fixes two equivalents of water, and then plays the part of an acid to alkaline bases. The salts thus formed are far more soluble than cantharidin, and are soluble in other menstrua, but these solutions are still very dilute when saturated, and appear feeble and sluggish in vesicating effect when compared with the activity of the oil. All this inclined the writer, after much labor, to go back to the beginning, and by the light and general drift of these trials, seek for some definite liquid representative of the crude drug like a fluid extract, wherein the active principles, as in fluid extract of ergot, may be rendered more fixed and permanent by new combination. After preliminary trials of various menstrua for exhausting the powdered cantharides, the officinal diluted alcohol, containing one-sixteenth of its volume of officinal liquor potassæ, seemed to succeed well. But when the liquid obtained and the residue after exhaustion were tried, the first was found rather sluggish in action, and loaded with an unnecessary amount of inert extractive matter, whilst the residue produced some irritation of the skin, and persistent itching after eighteen hours' application. After standing three weeks, the liquid was covered with a film of wax and fatty matter, and had become turbid. All this was accepted as evidence that the menstruum did not contain sufficient potassa and alcohol, and a new menstruum was made containing one-eighth of its volume of liquor potassæ, one-eighth water, and three-fourths stronger alcohol. This menstruum applied to the powder by repercolation yielded a percolate, which, when in the proportion of a minim for each grain of powder, was very active in producing vesication. Even when diluted to one-half this strength, its action was energetic and prompt, producing a blister in less than six hours. This percolate contained no free potassa, and the alcohol appeared to be in sufficient proportion to hold all the oil in solution. On standing exposed for some days it becomes turbid, and a film of waxy matter formed on the surface. This turbidity and waxy matter did not disappear on the addition of alcohol unless the liquid was heated. The percolate was miscible with water and with alcohol in all proportions, but with turbidity from the separation of waxy matter, with perceptible separation of oil. This percolate was accepted as the blistering liquid sought after. It is really a fluid extract of cantharides in the normal proportion of minim for grain, and as an example of how fluid extracts may be made by weight, and without measures, the formula by which it is practically made will now be given.

FLUID EXTRACT OF CANTHARIDES OR BLISTERING LIQUID.

Take of—

Cantharides, in fine powder, 16 troy ounces.

Solution of Potassa, }

Stronger Alcohol, } of each a sufficient quantity.

Water, }

Make a menstruum consisting of two troy ounces and thirty-two grains (= 2 f℥) of solution of potassa, and four troy ounces and three hundred and twenty grains (= 6 f℥) of stronger alcohol, to moisten the powder with.

Make another menstruum with which to percolate, consisting of 6¼ troy ounces of solution of potassa, 6 troy ounces of water, and 28 troy ounces of stronger alcohol. Divide the powdered cantharides into four equal parts, and moisten each part as it is wanted for percolation with one-fourth of the first menstruum. Then percolate the first part to exhaustion with the second menstruum. Reserve the first troy ounce of the percolate from this first part, and receive the remainder of the percolate in separate portions of about two or three troy ounces each. Repercolate the second part of the moistened powder with the percolate from the first part, excepting the reserved portion, and follow the percolate from the first part with about two troy ounces of new menstruum. Reserve three troy ounces of the first of the percolate from this second part, and separate the remainder of the percolate in fractions as before. Repercolate the third part of the moistened powder with the percolate from the second part, excepting the reserved portion, and follow the percolate with fresh menstruum as before. Reserve five troy ounces of the first of the percolate from this third part, and separate the remainder of the percolate in fractions as before. Repercolate the fourth and last part of the moistened powder with the percolate from the third part, excepting the reserved portion, and follow the weak percolate with fresh menstruum to exhaustion as before. Reserve five troy ounces of the first of the percolate from this fourth part, and separate the remainder of the percolate in fractions as before, to be carried on to a future process. Add the reserved portions of percolate together, and mix them as the finished fluid extract. When a new process is to be undertaken, four troy ounces of the powder is to be treated in exactly the same way as each of the above parts, and three and a half troy ounces of the first part of the percolate is to be reserved as finished fluid extract, and the remainder of the percolate is to be carried on to a future operation as before.

A pint of the first menstruum, at 25° C.= 77° F., weighs 6520 grains, and a pint of the second menstruum, at the same temperature, weighs 6482 grains. A pint of the finished fluid extract, at the same temperature, weighs 6850 grains, or 14 troy ounces and 130 grains. The normal difference for this lot of powdered cantharides is therefore 6850 —6520=330 grains.

The officinal liquor potassæ always contains lime and silica, which are precipitated upon admixture with alcohol, and these weights will vary from these figures if this precipitate be left in the mixture. As the officinal liquor potassæ contains 5.8 per cent. of hydrate of potassa, and as each fourteen troy ounces of the fluid extract contains about two troy ounces and thirty-two grains of liquor potassæ, it follows that the total quantity of hydrate of potassa represented in the fourteen troy ounces is about 57.5 grains or 0.85 per cent. This is, however, entirely saturated by the cantharidin and by the oil, and is present in the fluid extract as cantharidate of potassa and as a soap, both being held in perfect solution by the alcoholic menstruum, the turbidity on standing and by dilution consisting of inert matter in inconsiderable quantity. The only thing which appears undetermined in the preparation is its permanence. All the published researches on this point and all the analogies that are available, however, are in favor of its permanency, and as it proved very prompt and effective when reduced one-half in strength, it has a very good margin to secure its certainty.

It is therefore believed to be an excellent fluid extract of cantharides, and available for all therapeutic uses of the drug, internal as well as external, while it is both convenient and economical. It is, of course, an active poison, and should therefore bear a red label, and be dispensed with due care. As it can only be needed in small quantities, it should be put up in one and four ounce bottles.

For internal use it may be diluted to any desired extent, and may be given in any simple vehicle.

As a rubefacient, or mild and continuous counter-irritant, it may be added in very definite proportion to plasters, cerates, ointments, liniments, &c., to obtain any desired degree of effect.

As a vesicant, it is perhaps best applied by means of thin bibulous paper, such as common filtering paper or newspaper, covered by oiled silk or adhesive plaster. One minim of the fluid extract sufficiently moistens one square inch of such paper, and this is a good proportion in use,—a fluid drachm for an eight inch blister for example. The paper should be cut of the shape and size required, and a piece of oiled silk or oiled muslin, or a piece of common adhesive plaster, of the same shape but larger size, should be cut as a covering. The paper is then folded upon itself several times, and while lying on the middle of the oiled silk the fluid extract is dropped upon it until the paper is just thoroughly moistened or saturated, so that none of the liquid can drain out and spread over more of the surface than is desired. If it be accidentally or incautiously wetted too much, a few minutes' exposure will remedy this, by the evaporation of the alcohol. Paper thus moistened may be entirely dried, and be preserved for use in the dry state, to be moistened with water as required for use, but although likely to be more permanent, as shown by M. Delpech, than any of the common blistering tissues, its permanency has not yet been ascertained. It is therefore, for the present, considered better and safer to keep the blistering substance in the liquid form, and to moisten the paper with the liquid as it is required for use. This paper, thus moistened, is simply applied to the skin and covered with the oiled silk. In dispensing a blister of this kind, it is simply necessary to wrap the folded moistened paper up in the oiled silk, and then the whole in paper, with written directions for the simple mode of application. The skin should be cleansed from secretions before the paper is applied, but this is less necessary with this than with the ordinary forms of cantharides, because the active principles are here in a far more soluble condition. Of course, some means must generally be taken by bandage or by light compression to keep the paper in contact with the skin. A gentle pressure over the oiled silk for a few minutes, until it becomes warm and soft, will cause its margin to adhere to the surface around the paper, and usually this will be all that is needed. Narrow strips of adhesive plaster or isinglass plaster or a bandage may occasionally be required. Three to five hours' contact is generally sufficient to produce full vesication, but the cuticle will not generally be raised into a blister for six or seven hours, whether the paper be left in contact or not. The common practice of removing the blister at the end of five hours and substituting a water-dressing is excellent practice. If the blister be very painful and irritating to a patient, the water may be made with a half per cent. solution of carbolic acid, as in treating a superficial burn, instead of simple water, when the pain and soreness will promptly subside. This dressing, however, will lessen the counter-irritant effect of the blister somewhat, and to that extent will interfere with its object.

A collateral advantage of this fluid extract may be expected from the circumstance that the active principles are soluble in water, and may be easily washed from the surface by simple sponging after the blister is removed, thus lessening the absorption and the liability to strangury. It is also easily washed off the hands and other parts to which it becomes accidentally applied, and from the surface to which it is purposely applied, at any stage of its action. This should give it a great advantage over the cantharidal collodion in many of its applications, and should nicely adapt it to use about the os uteri. It should also be far more certain than the cantharidal collodion.

The writer regards this preparation as a striking example of the conservative ground which he has taken for some years past, that the search for, and the isolation of so-called active principles of drugs in therapeutics and pharmacy, may be easily, if it be not generally, carried too far. He believes that he has proved by the labor which is epitomized in this paper, that a well-constructed fluid extract of cantharides is in every practical sense better than any preparation of cantharidin, or of cantharidate of potassa, and is all-sufficient for every known use of the drug which it represents.

Therefore, as good fluid extract of ergot is far better for practical use than any of the so-called active principles of ergot; and as fluid extracts of aconite root, belladonna root, conium seed, and nux vomica, are better for therapeutic uses than aconitia, atropia, conia, and strychnia, so is a fluid extract of cantharides better than the drug itself, and better than its active principles. At least such is the conviction of this writer.

Treatment of Venereal Ulcers by Dr. Hemard.—In the *Med. Chirurg. Centralbl.*, 1871, Dr. Hemard asserts that for the last twenty years he has obtained the cure of soft and hard sores by simply irrigating the parts with cold water. A vessel of cold water is fixed to the walls of the room, at such a height a tube attains a certain force of projection. The patient has no more to do but to wash his ulcers every three or four hours under this stream; in a few days the ulcer becomes clean and quickly heals. All other treatment is superfluous. In ulcers of the prepuce, which are out of sight, after irrigating, a little starch flour is introduced. When the superficies of the ulcer has lost its characteristic aspect, a stratum of collodion is painted on it, and it soon heals.—*Id.*

### NOTE ON PAREIRA.

BY EDWARD R. SQUIBB, M.D.

Pareira Brava is a drug which has withstood the mutations of therapeutics and commerce for nearly two hundred years, and it is a singular and significant fact, in view of its commercial history, that it has sustained a sound reputation with many critical observers.

It appears to have been introduced to European practice from Portugal, but its sources were Mexico, tropical South America, and the West Indies. Under a name so indefinite as "wild vine," or "bastard vine,"—the translation of the name Pareira Brava,—it is hardly possible that the markets should have always been supplied from the same plant, even after its botanical source was determined, and hence the varying descriptions of different authorities may be accounted for. The writer has been familiar with it, both in its use and in its market character, for more than twenty-five years, and for the last half of this period supposed he knew the substance with some degree of accuracy, as its appearance was more uniform than that of most drugs. It, however, never had more than a very general agreement with any of the descriptions given of it; and the almost universal testimony of those physicians who knew it best was, that although very efficient in the treatment of chronic diseases of the mucous membranes of the urinary passages, it was only useful when given in doses very much larger than those prescribed by the books.

It has so happened, that in the New York market the trade in this drug has been largely, though not exclusively, confined to one drug house, and its appearance, as met with here, is identical with occasional samples seen from other cities. Some ten years ago, the annual sales here did not exceed three or four hundred pounds, and the price was fifteen to twenty cents. A Portuguese merchant, stimulated by this high price, imported a lot of some ten thousand pounds, and unable to sell it except in small lots at the expected prices, stored it for a year or two. This was found to be expensive management of so bulky an article, and the lot was finally sold at eight cents, and supplied the market for years. Another lot of about half as much shared the same fate, and fell into the same hands. The fate of these two lots and the glut of the market, seems to have stopped importation entirely, and by 1871, when the annual sales had reached three to four thousand pounds, the supply became exhausted. In resorting to foreign markets it was found scarce, and to be had only in small lots, and these, on arriving here, were held at seventy-five cents to a dollar a pound. In looking critically through one of these small lots as a purchaser, the writer was surprised to find nearly one-half of it so entirely different from any hitherto seen, that he rejected it, and at once pronounced it a fraudulent adulteration or substitution, made in the interest of the scarcity and high price, and carefully selected out for purchase that only which he had seen before. Some specimens of this supposed fraudulent Pareira were, however, taken for examination, and were found to agree well with some of the older descriptions. A plate given by Pomet in his History of Drugs, published in 1737, and a close examination of the structure, &c., convinced the writer that this was the true Pareira root, and that what he had heretofore seen was the stem.

In a critical review of the descriptions of Wood and Bache, and Pereira, these descriptions were found to apply to both, as nearly as such descriptions generally do to foreign drugs, but that they applied much better to the ligneous woody stem, which is comparatively insipid and inert. The root is very much darker, almost black externally, and both the annular and vertical wrinkles are very much larger and more prominent. It occurs in shorter sections than the stem, and gnarled pieces are found eight inches to a foot in diameter. The texture is far less compact than that of the stem, while the beautiful arrangement of the consecutive rings seen in a cross section, which requires a glass in the compact stem, is well seen with the naked eye in the root. The sweetish and afterward bitter taste of the woody stem is very feeble, and even when in the finest powder, it yields very little extract to any menstruum. The taste of the root is, however, very much stronger, and yields at least twice as much extractive matter to the menstrua. The specimens herewith presented illustrate the difference between the root and stem much better than any description, and will render further explanation unnecessary.

It thus appears that, for some twelve or fifteen years past, this market has been supplied with the comparatively inert stem, instead of the root of Pareira; and that the ideas of at least one careful purchaser had become so fixed upon the intractable woody stems, that when the roots did appear, they were very nearly rejected as a fraudulent substitution. The importations of this year thus far have come from the European markets in small lots, and have been a mixture of root and stem, but less of the root than stem, and the chief object of this note is to attract attention to the drug, and create such a demand for the proper root portion, that after the present scarcity is over, and the market comes to be again supplied direct, the stem may be rejected.

There is no doubt whatever as to the peculiar efficacy and utility of this drug within its legitimate sphere in therapeutics, and the wonder is that it has been able to sustain its well-tried and time-honored reputation upon the feeble medicinal properties of the stem.

---

Enuresis.—Dr. Woodman, in a clinical lecture at the London Hospital, published in the *Medical Press*, recommends the following method of investigating cases:—

1st.—Inquire carefully into the duration and frequency of the incontinence, and into the previous history of the child and the family.

2d.—Carefully note the diathesis, and general bodily condition of the patient.

3d.—Examine the *urine* by test papers, by other tests, and by the microscope if necessary.

4th.—Make a careful examination of your patient, including the anus and rectum amongst the pudenda, and, if need be, exploring the bladder also. I need not enjoin upon you all possible delicacy and tenderness in these examinations. You must explain the necessity to the parents—and you will often astonish them by evidences of neglect of cleanliness, or of the existence of sores of which they were ignorant.

5th and lastly.—Suit your treatment to the case; removing or soothing all causes of irritation, and at the same time endeavoring to improve the general health, for it is quite true that delicate and tubercular children, or those weakened by long and severe illness, such as the exanthemata, suffer most. Above all things *do not countenance corporal punishment for this* bodily infirmity. It is as cruel as it is usually useless; and I am in the habit of saying that the parents or nurses ought to be beaten, and not the little patients.

Dr. W. mentions the following *auxiliaries* to treatment, useful in a great number of cases:

There is first the warm-bath; a sitting-bath is all that is generally needed. It should not be too warm, indeed 90°, or at all events 98°, is quite warm enough. Next, I have found it of great service to forbid the little patients to drink anything after their tea (say at five or six o'clock), unless thirst be extremely pressing.

On the importance of *waking* them up once, twice, or more in the night to make water, I need not dwell. This is not so cruel as it seems; the child is soon asleep again in most cases. It is also well not to let them lie on the back. The trigone of the bladder is the most sensitive part, and, besides, it is possible the posterior regions of the spinal cord get unduly congested. A reel (such as used for cotton) fixed in the middle of the back sometimes prevents this, and the child may be turned over on the side. For local sores, the glycerine of tannin, aided by cleanliness, is very valuable. I wish it did not stain the sheets so much. As tonics, mineral acids with bark, and the perchloride of iron, are the best I know; and, if sweetened, children seldom refuse to take them, which is a great advantage.

As a sedative, belladona is, perhaps, often better than opium. You may give from one-eighth to one-fourth of a grain of the extract even to very young children, three or four times a day. This is the most successful empirical remedy I know, and obscure cases always deserve a trial, increasing the dose within the limits of safety. Should the urine, however, be saccharine, ammoniacal, or phosphatic, opium must have the preference. For ascarides you will use local injections. Salt and water enemata are as good as anything else. In epileptic cases, cod-liver oil (if the fits are tubercular in origin) will perhaps deserve to be considered as more than an auxiliary.—*Id.*

Of Drains, Smells, and Fevers.—The free ventilation that has recently been given to the unsavory subject of sewers and drains, in connection with outbreaks of typhoid fever, will, it is to be hoped, lead to practical ventilation of the sewers themselves, so that the noxious gases pent therein, and panting for escape, will be accommodated in this respect in a way as little prejudicial as possible to the health of the community. Time was when main drainage was unknown; houses were drained into cess-pits, and from time to time a functionary known as a "nightman," visited the premises in the dead of night, and bucketed out all the collected filth into his cart, to be carried away and used as manure. It did not appear that these nightmen were specially liable to fever or other disease in consequence of their avocation; indeed, the belief among some was that the occupation was rather healthy than otherwise, perhaps in consequence of the stimulating ammoniacal odors so freely inhaled.

However, we have now changed all this; at least, in most large towns; and houses are put in connection with an almost endless vista of long drains, which, while rather carriers than reservoirs of sewerage matter, are always full of most poisonous gas.

How many outbreaks of typhoid fever, especially in windy watering-places, have been called by the current of wind up these sewers, forcing the gases into houses, it is needless to say. One well-known instance we can easily call to mind, and the effect, at the suggestion of a well-known engineer, of introducing ventilators protected by charcoal, was a prompt means of thoroughly arresting further outbreak of typhoid in the place.

A consideration of these matters of experience will show that flushing the sewers of any house or district, at a moderate elevation above the trunk sewer, must be most dangerous, for the reason that, as the torrent of water rushes along, it displaces the gases that are collected in the sewer, and these, rising upwards, may be driven into a dwelling-house with all the force that is exercised by the pressure of the current of water.

It is, I presume, to guard against this very evil that Mr. Rawlinson has suggested the simple but most rational expedient of having the lid of every water-closet perforated with a hole just over the handle, so that this may be pulled up while the lid is closed, and so the stinking gases, displaced by the water-flow, cannot enter the house.

It would be out of place here to enter into the arrangements by which house-drains can be safely flushed. The principle to be kept in view is that the gases rise up as the water goes down; and such arrangements in the way of ventilation must be made as will secure for these gases a free outlet into the open air. The old-fashioned cess-pool of stagnant filth may have its objections; but practice tends to prove that sewerage in active motion may be more dangerous to the public health than the same matter stagnant and at rest.—*The Doctor.*

